# Esterification of phenyl acetic acid with *p*-cresol using metal cation exchanged montmorillonite nanoclay catalysts

**DOI:** 10.1098/rsos.171378

**Published:** 2018-02-07

**Authors:** M. Bhaskar, M. Surekha, N. Suma

**Affiliations:** 1 Department of Chemistry, Global Academy of Technology, Bengaluru, Karnataka, India; 2 Department of Chemistry, The Oxford College of Engineering, Bengaluru, Karnataka, India

**Keywords:** nanoclays, organic synthesis, green chemistry, catalysis

## Abstract

The liquid phase esterification of phenyl acetic acid with *p*-cresol over different metal cation exchanged montmorillonite nanoclays yields *p*-cresyl phenyl acetate. Different metal cation exchanged montmorillonite nanoclays (M*^n^*^+^ = Al^3+^, Zn^2+^, Mn^2+^, Fe^3+^, Cu^2+^) were prepared and the catalytic activity was studied. The esterification reaction was conducted by varying molar ratio of the reactants, reaction time and catalyst amount on the yield of the ester. Among the different metal cation exchanged catalysts used, Al^3+^-montmorillonite nanoclay was found to be more active. The characterization of the material used was studied under different techniques, namely X-ray diffraction, scanning electron microscopy and thermogravimetric analysis. The product obtained, *p*-cresyl phenyl acetate, was identified by thin-layer chromotography and confirmed by Fourier transform infrared, ^1^H NMR and ^13^C NMR. The regeneration activity of used catalyst was also investigated up to fourth generation.

## 1. Introduction

Nanoclays have been one of the significant industrial minerals and with the recent development of nanoclay technology. Montmorillonite, {[M_2_(OH)_2_(Si_4_O_10_)]·*x*H_2_O}, M = Al and/or Mg is one of the most important nanoclay minerals used in various organic reactions [1]. Clay nanoparticles are layered structures and a layer possesses negative charge that is neutralized by many cations [2]. Montmorillonite nanoclays have one octahedral sheet containing aluminium or magnesium sandwiched between two tetrahedral sheets containing silicon [3]. Montmorillonite has the capability to exchange various metal cations like Al^3+^, Zn^2+^, Mn^2+^, Fe^3+^,Cu^2+^, Cr^3+^, Ni^2+^ etc. by the cations present in the interlayer of nanoclay mineral [4]. The catalytic activity of nanoclay mineral has been enhanced by manipulating the pore size, intercalating and replacing interlayer cations and surface area [5].

Nanoclay mineral is considered as the green catalyst for several important reactions to synthesize fine chemicals. They found applications in several fields like cosmetics, catalysis, food packaging and also in medicine [1,2]. Modified montmorillonite nanoclays are used to catalyse various organic transformations such as addition, condensation, oxidation of alcohol, formation of aldols, acylation, allylation, hydrogenation, dehydrogenation, alkylation, heterocyclic synthesis, esterification, rearrangement, cyclization and many more [6,7]. Nanoclay minerals are ecofriendly, non-corrosive, non-toxic, abundantly available, low-cost chemical and easily modified materials. Because of larger surface area, great ion-exchange capacity, high selectivity and reusability, the nanoclay is considered as prominent and evergreen catalyst in the field of catalysis [8,9].

Modified nanoclay catalyst provides ecofriendly substitute for Brønsted acids such as concentrated sulfuric acid or nitric acid and alternative for Lewis acid like AlCl_3_. Montmorillonite has been reported as effective acid catalytic substance for the organic reactions such as dicarboxylic acids to aromatic anhydrides [10], dimerization of ethylene oxide to dioxygen heterocycles [11] and ether synthesis [12]. The present study attempts to explain the esterification of phenyl acetic acid (PA) with *p*-cresol (*p*-C) using intercalatable metal cation (M*^n^*^+^ = Al^3+^, Zn^2+^, Mn^2+^, Fe^3+^, Cu^2+^) exchanged nanoclays. A significant improvement in the esterification reaction corresponds to phenyl acetate ester with higher yield. Esters are important organic compounds having applications in industries in the production of perfumes, flavours, plasticizers, cosmetics, solvents, pharmaceuticals and intermediates [13].

The study reviews the preparation of different metal cation exchange nanoclays and liquid phase esterification of PA with *p*-C to yield *p*-cresyl phenyl acetate in different experimental condition. The reactivity of nanoclay catalyst has been studied up to fourth generation in order to describe the regenerated activity. The *p*-cresyl phenyl acetate is used in industries as floral chemicals in perfumes such as lily, narcissus, hyacinth and jasmine and in floral soaps [14].

## 2. Material and methods

### 2.1. Materials

The nanoclay mineral used in this study is a montmorillonite K-10 powder procured from Sigma-Aldrich, Bangalore, India. The chemicals PA, *p*-C, solvents (toluene, benzene, chlorobenzene, 1,4-dioxane) and substituted phenols (*m*-cresol, *o*-cresol, *p*-nitrophenol, *m*-nitrophenol, *p*-methoxy phenol, *p*-tert-butyl phenol) were purchased from SD Fine Chemicals, Mumbai, India. The *p*-C, solvents and substituted phenols were distilled before use.

### 2.2. Preparation of metal cation exchange nanoclays (M*^n^*^+^–mont-nanoclay)

The method involves stirring raw montmorillonite nanoclay overnight with 0.5 M (200 ml) different metal cation solutions (M*^n^*^+^ = Al^3+^, Zn^2+^, Fe^3+^, Cu^2+^, Mn^2+^ etc.) to get M*^n^*^+^–mont-nanoclay catalyst. Then the clay was centrifuged and washed repeatedly with distilled water until the washings were free from chloride ions. This was confirmed by silver nitrate test. The M*^n^*^+^–mont-nanoclay sample was dried at 100°C for 30 min and finely powdered [15,16].

### 2.3. Catalytic study

**Figure 1.**
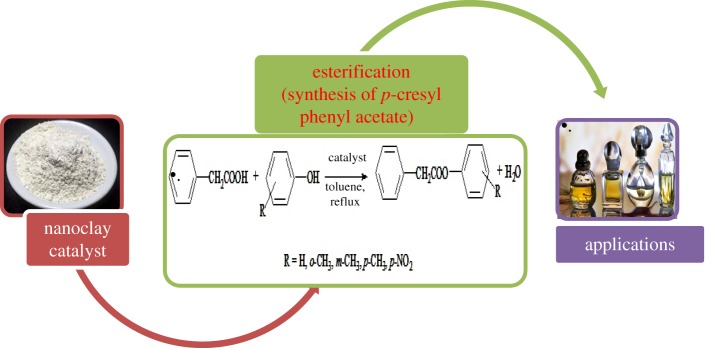
Overview of esterification of PA using metal cation exchanged nanoclays.

The esterification reaction of PA with *p*-C by using suitable solvent was carried out in 100 ml of round bottom flask fitted with a reflux condenser. The esterification study was conducted by refluxing 25 mmol of PA, 50 mmol of *p*-C and 0.5 g of prepared M*^n^*^+^–mont-nanoclay catalyst using 30 ml of toluene as a solvent (figure 1). The reaction was refluxed, cooled and filtered to separate M*^n^*^+^–mont-nanoclay catalyst and washed twice with solvent. The filtrate was treated with 5% NaOH in separating funnel to remove unreacted reactants followed by water and saturated brine solution. The solvent was distilled off from organic layer under reduced pressure and dried over anhydrous sodium sulfate [15,16]. The resulted product, *p*-cresyl phenyl acetate, was extracted with diethyl ether and this was identified by thin-layer chromatography and confirmed by Fourier transform infrared (FTIR) (Perkin Elmer, Spectrum Two, 100300), ^1^H NMR (Brucker, 400 MHz) and ^13^C NMR (Brüker-AMX 400).

### 2.4. Mechanism of esterification

**Figure 2.**
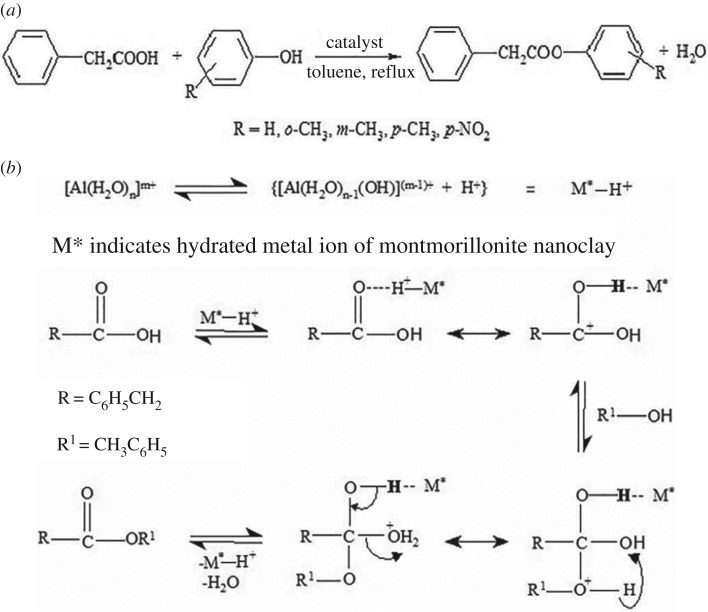
(*a*) Schematic representation of esterification of phenyl acetic acid with phenols over M*^n^*^+^–mont-nanoclay catalyst. (*b*) Possible mechanism of esterification of PA with *p*-C over M*^n^*^+^–mont-nanoclay catalyst.

The mechanism of esterification of PA with *p*-C in the presence of M*^n^*^+^–mont-nanoclay catalyst is as shown in figure 2*b*. Esterification reaction between PA and alcohol is known to be catalysed by Brønsted acid sites. PA gets protonated at the Brønsted acid sites and forms conjugate acid ion, i.e. oxonium ion. The addition of alcohol to the oxonium ion forms an intermediate compound which on loss of proton and water leads to formation of the ester.

### 2.5. Spectral studies

**Figure 3.**
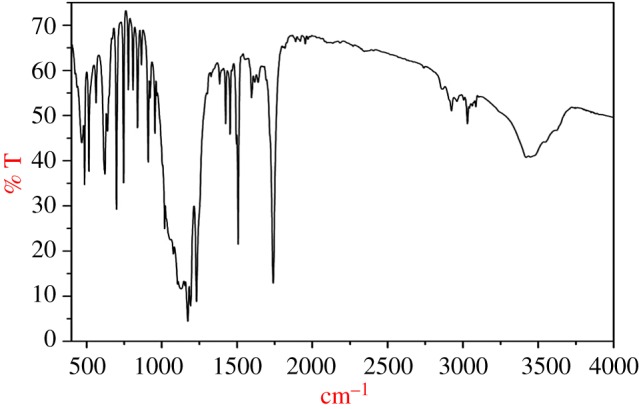
FTIR spectrum of *p*-cresyl phenyl acetate. The IR spectra at 1742 cm^−1^ clearly stated the formation of ester on esterification of PA using M*^n^*^+^–mont-nanoclay catalyst.

**Figure 4.**
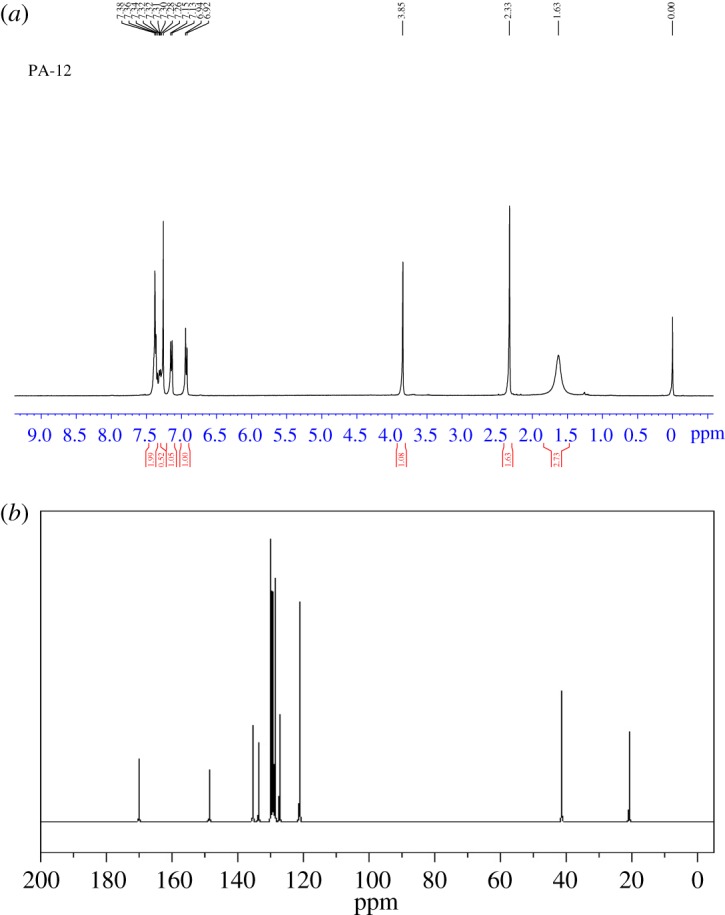
(*a*) ^1^H NMR spectrum of *p*-cresyl phenyl acetate. ^1^H NMR data of *p*-cresyl phenylacetate (*δ *= ppm): 2.33 (s, 3H), 3.85 (s, 2H), 6.92 (d, 2H), 7.15 (d, 2H) and 7.30–7.38 (m, 5H). (*b*) ^13^C NMR spectrum of *p*-cresyl phenyl acetate (*δ *= ppm) 170.0, 148.6, 135.3, 133.6, 129.8, 129.3, 128.7, 127.2, 121.1, 40.8, 20.7.

FTIR data (figure 3) of *p*-cresyl phenyl acetate (cm^−1^): 3027, 2925, 1742, 1506, 1401, 1229, 1161, 908, 840, 618 and 495. IR peaks obtained at 1742 cm^−1^ are due to C = O group (ester), peak at 1506 cm^−1^ indicates C–O stretching, peak at 2925 cm^−1^ represents C–H stretching and peak at 495 cm^−1^ indicates benzene ring having substitution. ^1^H NMR data (figure 4*a*) of *p*-cresyl phenyl acetate (*δ *= ppm): 2.33 (s, 3H), 3.85 (s, 2H), 6.92 (d, 2H), 7.15 (d, 2H) and 7.30–7.38 (m, 5H). ^13^C NMR data (figure 4*b*) of *p*-cresyl phenyl acetate (*δ *= ppm) 170.0, 148.6, 135.3, 133.6, 129.8, 129.3, 128.7, 127.2, 121.1, 40.8, 20.7.

### 2.6. Regeneration of catalyst

After the reaction, the M*^n^*^+^–mont-nanoclay was separated by filtration from the reaction mixture, dried and washed with distilled water. The M*^n^*^+^–mont-nanoclay was dried at 100°C at 30 min, powdered and used again for the esterification reaction to study its catalytic activity.

## 3. Results and discussion

### 3.1. Characterization of material

Specific surface area of raw montmorillonite nanoclay sample was determined by BET methods using a Quantachrome NOVA 1000 surface area analyser at liquid nitrogen temperature. The surface area of the raw montmorillonite nanoclay was found to be 230 m^2^ g^−1^.

**Figure 5.**
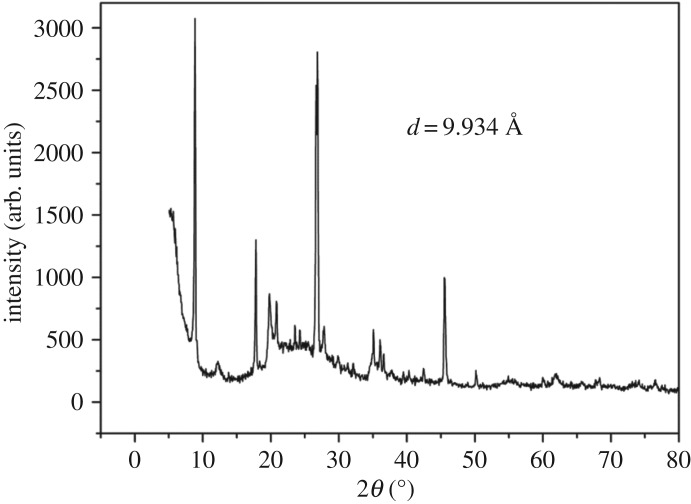
XRD pattern of raw montmorillonite nanoclay. In raw montmorillonite nanoclay, the interlamellar space will be accommodated by simple ions like Na^+^ and Ca^2+^ in their hydrated form. The simple ions of montmorillonite are exchanged with large species, which occupy the interlamellar space and expand it. This expansion of layers can be observed by XRD. The basal spacing of raw montmorillonite nanoclay was identified as 9.934 Å.

**Figure 6.**
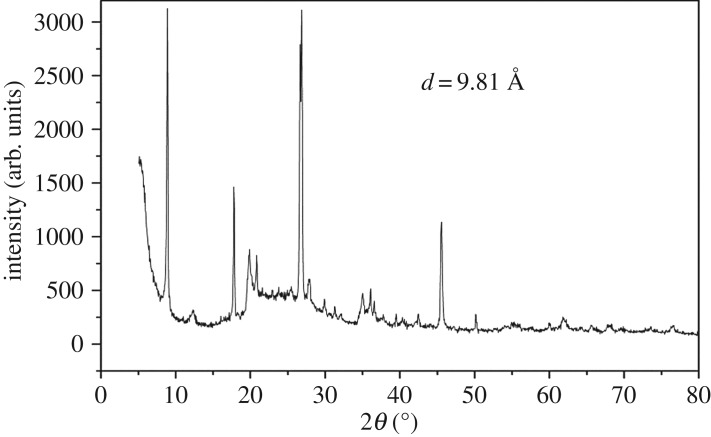
XRD pattern of Al^3+^–mont-nanoclay. The basal spacing for the Al^3+^–mont-nanoclay was identified as 9.81 Å, which is greater than the raw montmorillonite nanoclay material. This is clearly due to the presence of intercalatable Al^3+^ ions at interlamellar region.

The X-ray diffraction (XRD) pattern of the raw montmorillonite nanoclay and Al^3+^–mont-nanoclay were recorded using Siemens D5005 diffractometer using Cu–K*α* radiation source between 2*θ* values 3° and 40°. The basal spacing of the samples, raw montmorillonite nanoclay and Al^3+^–mont-nanoclay were identified as 9.934 Å (figure 5) and 9.81 Å (figure 6), respectively. XRD pattern clearly evidences that there is enhancement of basal spacing of Al^3+^–mont-nanoclay compared with raw montmorillonite nanoclay. The reason for this enhancement may be due to swelling of the interlayers by intercalation of Al^3+^ cation.

**Figure 7.**
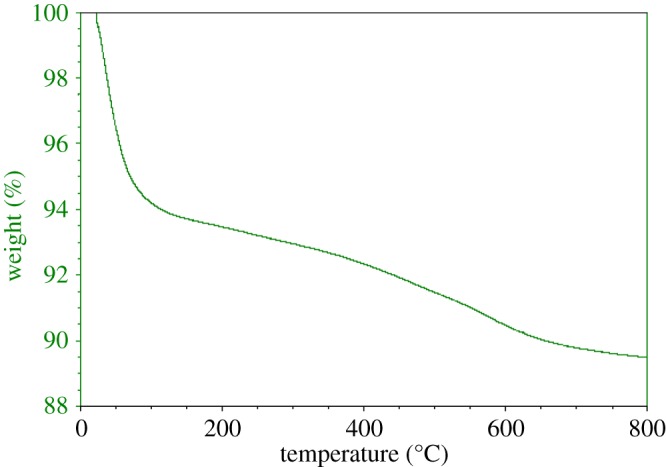
Thermogravimetric pattern of raw montmorillonite nanoclay. Weight loss below 120°C is believed to be due to loss of water and the weight loss between the temperature 120 and 600°C is due to loss of hydroxyl groups. The weight loss above 600°C observed to be negligible.

**Figure 8.**
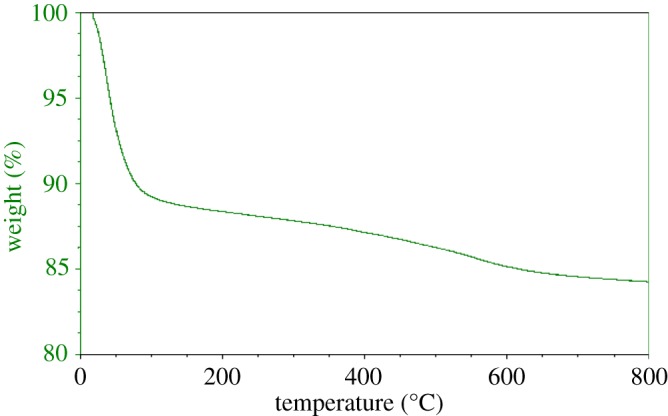
Thermogravimetric pattern of Al^3+^–mont-nanoclay. The loss of hydrated water occurs slightly at a lower temperature and the hydrated water more strongly held in modified clay due to the presence of high polar cations.

Thermogravimetric analysis (TGA) of the raw montmorillonite nanoclay (figure 7) and Al^3+^–mont-nanoclay (figure 8) were carried out using Universal V4.5A between the temperatures 30° and 800°C with heating range 10°C min^−1^. TGA curve of the raw nanoclay and Al^3+^–mont-nanoclay exhibits an initial sharp decrease due to loss of water and second one beyond 120°C due to loss of organic group. No significant change takes place above 700°C. TGA patterns of the raw montmorillonite nanoclay and Al^3+^–mont-nanoclay clearly stated that the materials used for the esterification reaction are thermally stable.

**Figure 9.**
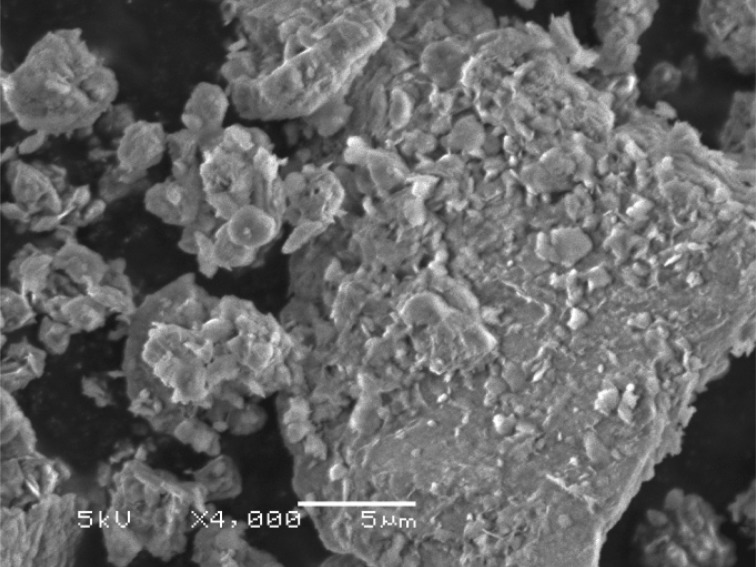
SEM image of raw montmorillonite nanoclay at a magnification of 4000×.

**Figure 10.**
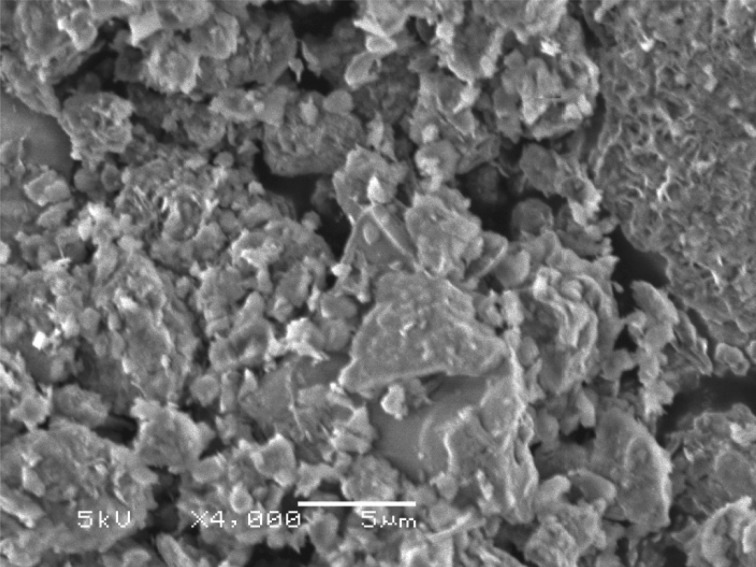
SEM image of Al^3+^–mont-nanoclay at a magnification of 4000×.

Scanning electron microscopy (SEM) images of raw montmorillonite nanoclay (figure 9) and Al^3+^–mont-nanoclay (figure 10) were captured using JEOL JSM-840A instrument. SEM images indicate that surface area of raw montmorillonite nanoclay is more than the Al^3+^–mont-nanoclay.

### 3.2. Effect of molar ratio

The molar ratio of the reactants such as PA and *p*-C was varied between 25 and 80 mmol to get different molar ratios. The reaction was refluxed for 6 h using 0.5 g of M*^n^*^+^–mont-nanoclay catalysts. The yield of the product increased and reached a maximum of 58% when the molar ratio of PA and *p*-C was 1 : 4. The percentage yield obtained in different molar ratio of the reactants is given in table 1.

**Table 1.** Effect of molar ratio on the yield of *p*-cresyl phenyl acetate. Reactant, PA : *p*-C (25–80 mmol); reaction time, 6 h; amount of catalyst, 0.5 g; catalyst used, M*^n^*^+^–mont-nanoclay catalyst; solvent, toluene (30 ml).

	yield of *p*-cresyl phenyl acetate (%)				
molar ratio (phenyl acetic acid : *p*-C)	Al^3+^	Zn^2+^	Mn^2+^	Fe^3+^	Cu^2+^
1 : 1	19	07	nil	07	04
1 : 2	06	01	nil	05	02
1 : 3	12	04	nil	08	06
1 : 4	58	40	11	20	15
1 : 5	24	18	01	09	09
1 : 6	19	10	06	04	04
2 : 1	11	12	03	03	02
3 : 1	18	15	06	05	04
4 : 1	22	16	09	07	05
5 : 1	14	10	05	05	04
6 : 1	08	07	03	04	03

### 3.3. Effect of reaction period

**Figure 11.**
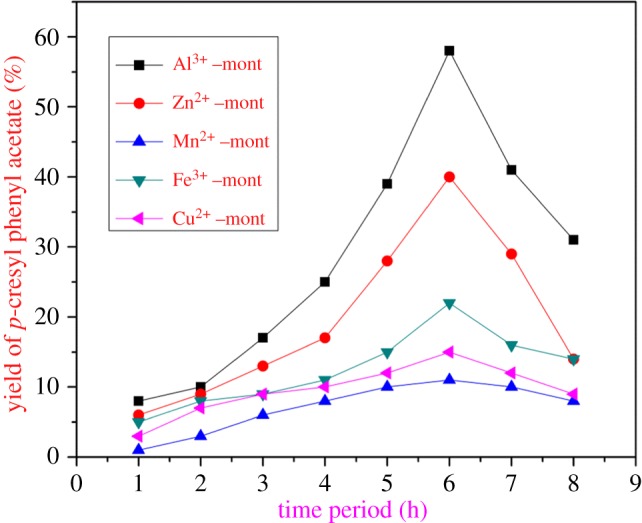
Effect of reaction period on the yield of the ester. Reactants, PA : *p*-C (1 : 4); amount of catalyst, 0.5 g; catalyst used, M^n+^–mont-nanoclay catalyst; solvent, toluene (30 ml).

The reaction was carried out at different time periods under the same experimental conditions. A series of reactions were conducted by refluxing PA with *p*-C (1 : 4 molar ratio), 0.5 g M*^n^*^+^–mont-nanoclay catalyst. The yield of the product increased as the increase in the reaction time from 1 to 8 h and there was a decrease in the ester yield when the reaction time reaches beyond 6 h. This is due to the shift in the equilibrium of catalysed esterification reaction. So, the optimum reaction period for the esterification of PA and *p*-C was reported as 6 h (figure 11).

Blank reaction was also conducted by refluxing PA and *p*-C (molar ratio = 1 : 4), 0.5 g montmorillonite raw nanoclay catalyst and solvent (toluene = 30 ml) for 6 h. It was reported that raw montmorillonite nanoclay failed to catalyse the esterification even after refluxing for 8 h.

### 3.4. Effect of catalyst amount

**Figure 12.**
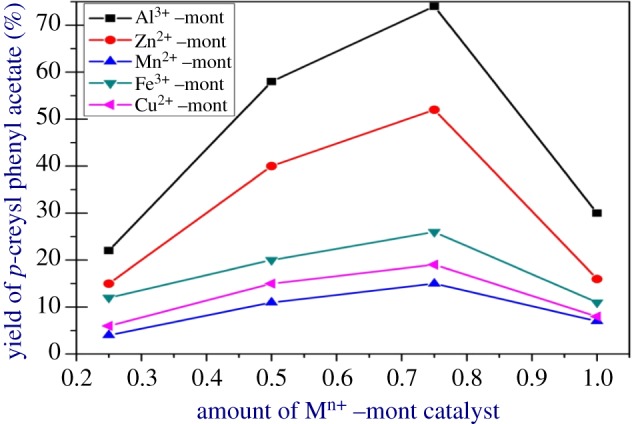
Effect of catalyst amount on the yield of the ester. Reactants, PA : *p*-C (1 : 4); reaction period, 6 h; catalyst used, M*^n^*^+^–mont-nanoclay catalyst; solvent, toluene (30 ml).

The esterification reaction was conducted by varying the amount of M*^n^*^+^–mont-nanoclay catalyst in order to study the effect on the yield of the ester (figure 12). The amount of M*^n^*^+^–mont-nanoclay catalyst was increased from 0.25 to 1 g. It was reported that the yield of an ester increased with increase in amount of M*^n^*^+^–mont-nanoclay catalyst and the maximum yield was obtained when the catalyst amount was 0.75 g used. The increased amount of catalyst increases the yield of the ester due to the increase in the number of acid sites available for esterification reaction. For catalyst amounts above 0.75 g the yield of the ester was found to be decreased, which is apparently due to the availability of excess active sites on the catalyst surface for esterification leading to the decomposition of the product.

### 3.5. Activity of regenerated catalyst

The catalytic activity of regenerated M*^n^*^+^–mont-nanoclay catalyst was investigated after washing with distilled water and drying at 100°C. The esterification reactions were carried out under same experimental conditions. The obtained yield of product per unit mass was found to be nearly the same even after regenerating the M*^n^*^+^–mont-nanoclay catalyst five times as that yield obtained using fresh catalyst. The study evidenced the presence of active sites on reused M^n+^–mont-nanoclay catalyst (table 2).

**Table 2.** Activity of regenerated catalyst. Reactants, PA : *p*-C (1 : 4); reaction period, 6 h; catalyst used, M*^n^*^+^–mont-nanoclay catalyst; solvent, toluene (30 ml).

	yield of *p*-cresyl phenyl acetate (%)									
	Al^3+^		Zn^2+^		Mn^2+^		Fe^3+^		Cu^2+^	
regeneration	amount (g)	yield	amount (g)	yield	amount (g)	yield	amount (g)	yield	amount (g)	yield
fresh	0.75	74	0.75	52	0.75	15	0.75	26	0.75	19
first time	0.72	71	0.70	48	0.72	11	0.71	21	0.70	14
second time	0.67	68	0.65	44	0.66	08	0.67	19	0.65	11
third time	0.50	65	0.53	41	0.60	06	0.58	15	0.54	09
fourth time	0.42	60	0.44	37	0.51	03	0.42	11	0.44	03

### 3.6. Effect of different solvents

The esterification of PA with *p*-C was carried out by using different solvents like toluene, benzene, chlorobenzene and 1,4-dioxane to investigate the effect of different solvents on the yield of the ester (table 3). The result of esterification with different solvents is that the percentage yield decreases with an increase in the polarity of the solvents. Non-polar solvent gives more yield than the polar solvent. The esterification reaction in different metal cations using toluene as a solvent was reported as the maximum yield compared with other solvents used. Because the polarity of toluene is less compared with other solvents used in the reaction. The relative polarity of the solvents like toluene, benzene, 1,4-dioxane and chlorobenzene are 0.099, 0.111, 0.164 and 0.188, respectively.

**Table 3.** Effect of different solvents on esterification. Reactants, PA : *p*-C (1 : 4); amount of catalyst, 0.75 g; reaction period, 6 h; catalyst used, M*^n^*^+^–mont-nanoclay catalyst.

	yield of *p*-cresyl phenyl acetate (%)				
solvents	Al^3+^	Zn^2+^	Mn^2+^	Fe^3+^	Cu^2+^
toluene	74	52	15	26	19
benzene	24	18	14	16	09
chlorobenzene	nil	nil	nil	nil	nil
1,4-dioxane	13	nil	07	nil	nil

### 3.7. Effect of substituted phenols on esterification

The esterification of phenyl acetic acid was also conducted with different substituted phenols using M*^n^*^+^–mont-nanoclay catalysts (table 4). The reactions were conducted keeping reaction conditions such as phenyl acetic acid to *p*-C mole ratio 1 : 4, catalyst amount 0.75 g, toluene solvent and reaction time 6 h. Among cresols, *p*-C gave the highest yield, whereas the *o*-cresol yields much less. This is possibly due to steric factors. The esterification with nitrophenols gave negligible yields, it is clear that the nitro group has a negative effect on the rate of esterification, because its electron-withdrawing property reduces the nucleophilic character of phenol.

**Table 4.** Effect of substituted phenol on esterification. Reactants, phenyl acetic acid : *p*-C (1 : 4); amount of catalyst, 0.75 g; reaction period, 6 h; catalyst used, M*^n^*^+^–mont-nanoclay catalyst; solvent, toluene (30 ml).

	yield of *p*-cresyl phenyl acetate (%)				
substituted phenol	Al^3+^	Zn^2+^	Mn^2+^	Fe^3+^	Cu^2+^
*p*-cresol	74	52	15	26	19
*m*-cresol	58	41	10	18	11
*o*-cresol	04	01	nil	nil	nil
phenol	51	35	07	11	05
*p*-nitrophenol	03	nil	nil	nil	nil
*o*-nitrophenol	nil	nil	nil	nil	nil
*p*-methoxy phenol	nil	nil	nil	nil	nil
*p*-tert-butyl phenol	nil	nil	nil	nil	nil

## 4. Conclusion

Montmorillonite nanoclay exchanged with different metal cations (M*^n^*^+^ = Al^3+^, Zn^2+^, Fe^3+^, Cu^2+^ and Mn^2+^) catalyses the esterification of PA with *p*-C. The percentage of yield obtained in esterification reaction using M^n+^–mont-nanoclay catalyst indicates that Al^3+^–mont-nanoclay has better catalytic activity compared with other metal cation exchanged nanoclays. The activity of the Al^3+^–mont-nanoclay is nearly equal to the catalyst obtained commercially. The percentage yield obtained in esterification using nanoclay catalyst is nearly equal to the conventional methods. The catalyst used has strong Brønsted acid sites to catalyse esterification reaction. The recyclability of these solid catalysts renders these processes economical. Other advantages of this method include operational simplicity, environmentally friendly and reusable nature of the catalyst.

## Data accessibility

The data are included in the manuscript in the form of tables and figures.
